# Strong mitochondrial DNA support for a Cretaceous origin of modern avian lineages

**DOI:** 10.1186/1741-7007-6-6

**Published:** 2008-01-28

**Authors:** Joseph W Brown, Joshua S Rest, Jaime García-Moreno, Michael D Sorenson, David P Mindell

**Affiliations:** 1 University of Michigan Museum of Zoology and Department of Ecology and Evolutionary Biology, 1109 Geddes Avenue, Ann Arbor, MI 48109-1079, USA; 2 Department of Ecology and Evolution, University of Chicago, 1101 East 57th Street, Chicago, IL 60615, USA; 3 Centre for Biodiversity Conservation Mexico and Central America, Conservation International, Apdo. 2365-2050 San Pedro, Costa Rica; 4 Department of Biology, Boston University, 5 Cummington Street, Boston, MA 02215, USA

## Abstract

**Background:**

Determining an absolute timescale for avian evolutionary history has proven contentious. The two sources of information available, paleontological data and inference from extant molecular genetic sequences (colloquially, 'rocks' and 'clocks'), have appeared irreconcilable; the fossil record supports a Cenozoic origin for most modern lineages, whereas molecular genetic estimates suggest that these same lineages originated deep within the Cretaceous and survived the K-Pg (Cretaceous-Paleogene; formerly Cretaceous-Tertiary or K-T) mass-extinction event. These two sources of data therefore appear to support fundamentally different models of avian evolution. The paradox has been speculated to reflect deficiencies in the fossil record, unrecognized biases in the treatment of genetic data or both. Here we attempt to explore uncertainty and limit bias entering into molecular divergence time estimates through: (i) improved taxon (*n *= 135) and character (*n = *4594 bp mtDNA) sampling; (ii) inclusion of multiple cladistically tested internal fossil calibration points (*n *= 18); (iii) correction for lineage-specific rate heterogeneity using a variety of methods (*n *= 5); (iv) accommodation of uncertainty in tree topology; and (v) testing for possible effects of episodic evolution.

**Results:**

The various 'relaxed clock' methods all indicate that the major (basal) lineages of modern birds originated deep within the Cretaceous, although temporal intraordinal diversification patterns differ across methods. We find that topological uncertainty had a systematic but minor influence on date estimates for the origins of major clades, and Bayesian analyses assuming fixed topologies deliver similar results to analyses with unconstrained topologies. We also find that, contrary to expectation, rates of substitution are not autocorrelated across the tree in an ancestor-descendent fashion. Finally, we find no signature of episodic molecular evolution related to either speciation events or the K-Pg boundary that could systematically mislead inferences from genetic data.

**Conclusion:**

The 'rock-clock' gap has been interpreted by some to be a result of the vagaries of molecular genetic divergence time estimates. However, despite measures to explore different forms of uncertainty in several key parameters, we fail to reconcile molecular genetic divergence time estimates with dates taken from the fossil record; instead, we find strong support for an ancient origin of modern bird lineages, with many extant orders and families arising in the mid-Cretaceous, consistent with previous molecular estimates. Although there is ample room for improvement on both sides of the 'rock-clock' divide (e.g. accounting for 'ghost' lineages in the fossil record and developing more realistic models of rate evolution for molecular genetic sequences), the consistent and conspicuous disagreement between these two sources of data more likely reflects a genuine difference between estimated ages of (i) stem-group origins and (ii) crown-group morphological diversifications, respectively. Further progress on this problem will benefit from greater communication between paleontologists and molecular phylogeneticists in accounting for error in avian lineage age estimates.

## Background

Many evolutionary models [1-4] are tightly linked to absolute timescales. A reliable temporal framework is therefore required for understanding the tempo (and, through correlation with geophysical phenomena, mechanisms) of biological evolution. There are two complementary sources of information for dating ancient biological divergences: (1) physical historical remains (either paleontological or ichnological); and (2) molecular sequence differences among extant taxa, the analysis of which requires assumptions about the processes and rates of sequence evolution. Unfortunately, these two sources of information ('rocks' and 'clocks', respectively) often yield starkly disparate estimates of the timing of major biological divergences [5].

**Figure 1:**
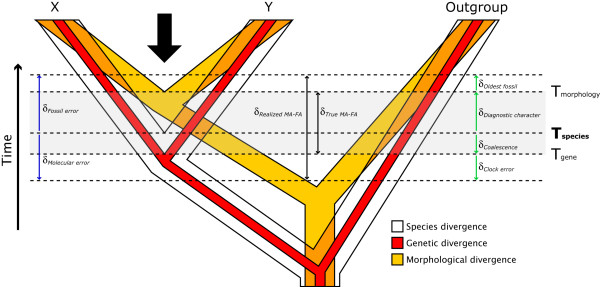
**Different ways that fossil and molecular data date lineages**. Time intervals defined by the horizontal dashed lines and vertical arrows pertain to age estimates for the divergence between hypothetical lineages X and Y. Even with a complete fossil record and perfect molecular clock a discrepancy is expected between fossil (FA) and molecular (MA) age estimates. As diagnostic morphological characters generally evolve (*T*_Morphology_) after species divergence (*T*_Species_), the fossil record will always underestimate (by δ_*Diagnostic character*_) the true speciation time. Genetic data, on the other hand, will overestimate speciation time (by δ_*Coalescence*_), as polymorphisms present during species divergence will coalesce some time in the past (*T*_Gene_; related to the ancestral species effective population size). The genuine difference between molecular and morphological divergence times will thus be δ_*True MA-FA*_. With a less complete fossil record, the oldest known fossil is unlikely to temporally correspond precisely to the origination of a diagnostic character delimiting X and Y, further decreasing FA by δ_*Oldest fossil*_. Under the more realistic scenario of lineage-specific rate heterogeneity and limited taxon/character sampling, errors associated with molecular methods (δ_*Clock error*_) may result in overestimation or underestimation of the true speciation time, although underestimates are bounded by the fossil constraint (δ_*Fossil error*_). The observed discrepancy in age estimates, δ_*Realized MA-FA*_, may be considerably larger than expectations (δ_*True MA-FA*_).

Of course, some discrepancy is expected, as these two sources of data concern different stages during the process of cladogenesis (δ_*True MA-FA*_; Figure 1). As fossils document products of evolution, they necessarily post-date speciation events. The underestimation of speciation times from fossil data (δ_*Fossil error*_) can be partitioned into two components: (i) the interval, following speciation, required for diagnostic characters to evolve (δ_*Diagnostic character*_); and (ii) the time, following the evolution of diagnostic characters, realized for the deposition of a sampled fossil (δ_*Oldest fossil*_). Here, δ_*Diagnostic character *_can be regarded as a fixed value (although different for every node), however δ_*Oldest fossil *_can be reduced with subsequently older fossil finds. In contrast to fossils, molecular data instead reflect genetic divergence, which must predate speciation events because genetic lineages present in two newly evolved sister lineages coalesce (on average) 2N_e _generations prior to speciation [6]. However, the errors associated with molecular age estimates (δ_*Coalescence *_and δ_*Clock error*_) are more complex than analogous fossil errors. For example, if no polymorphism exists for a particular locus at speciation, then inferred genetic divergence times based on that locus will actually post-date speciation, as no information exists to trace the underlying genealogy. Furthermore, molecular data may overestimate or underestimate the true speciation time because of stochastic errors associated with divergence time estimation (δ_*Clock error*_), and this uncertainty will increase as one extrapolates backwards through time, even with an infinite amount of data [7]. Regardless, for a given node with a minimum age constraint derived from the fossil record, the realized discrepancy between the two estimates (δ_*Realized MA-FA *_= MA - FA = δ_*Molecular error *_+ δ_*Fossil error*_) will always be positive, and is a parameter that both paleontologists and molecular biologists are working to minimize [5].

Strictly speaking, any molecular estimate that generates a positive value of δ_*Realized MA-FA *_is consistent with the fossil record. It is instead the *magnitude *of δ_*Realized MA-FA *_that suggests conflict, and distressingly large values sometimes exist. Conflict between the two sources of information is especially evident with respect to the ages of extant avian lineages (Neornithes). Based on a strict interpretation of the fossil record (i.e. insignificant δ_*Fossil error*_), Feduccia [8,9] proposed an explosive Cenozoic origin for most modern avian lineages, presumably a result of open niches left by newly extinct non-avian dinosaurs and other taxa. Although a recent fossil find [10] forces a minimum of five of the earliest Neornithes divergences into the late Cretaceous, the fossil record generally supports the view that most modern lineages originated in the Cenozoic [8,9,11-15]. In contrast, molecular estimates all indicate that these same lineages are considerably older, sometimes as much as twice as old as analogous paleontological estimates [4,16-26]. Between these two extremes lies the Cretaceous-Paleogene (K-Pg; formerly Cretaceous-Tertiary or K-T) boundary, a period when as many as 50% of land-dwelling species went extinct [27]. The conflicting age estimates thus have different implications regarding the influence of the K-Pg mass extinctions on the evolutionary radiation of modern birds.

Although resolution of this conflict is clearly important for understanding avian diversification, it is hindered by compelling arguments from both sides. The supposition that the quality of the fossil record deteriorates backwards in time, for example, is contradicted by the observed congruence between stratigraphic and phylogenetic ordering of taxa [28]. Sophisticated stratigraphic analyses indicate that fossils of the antiquity necessary to produce congruence with molecular studies are extremely improbable [11,29,30] (but see [31,32]). Furthermore, of the known Mesozoic avian fossils [12,14,33,34], the vast majority unambiguously lay outside Neornithes [35]. Although a few Cretaceous fossils putatively represent modern lineages (e.g. parrot [36], loon [37] and others [12,14]) these have largely been dismissed as fragmentary and inconclusive [9,12,38,39]. One the molecular side, the recognition that rates of molecular evolution are often not clock-like (including birds [23,40-42]), and that lineage-specific heterogeneity is common [43], has spurred the development of numerous 'relaxed' molecular clock methods (see reviews in [44-46]). In support of molecular genetic data, these methods perform well in simulation [47,48] and, when applied to empirical data, deliver Cretaceous ages for the origin of modern birds [16,23].

Given these arguments, the paleontological and molecular phylogenetic communities are currently at an impasse regarding the application of an absolute temporal axis for early organismal evolution [33,49], and a range of evolutionary models [1-4] remain viable for birds. Here we endeavour to determine whether a more rigorous treatment of molecular genetic data lessens the 'rock-clock' discrepancy (δ_*Realized MA-FA*_). In particular, we incorporate large fossil and taxon data sets, two components of molecular dating that have been shown to have a strong impact on the resulting divergence time estimates [50,51]. In addition, we accommodate and explore the impact of uncertainty in both tree topology and molecular dating strategy. Finally, we test for signals of episodic molecular evolution related to both speciation events and absolute geologic time, processes that could potentially mislead molecular-based age estimates by systematically inflating branch lengths within speciose clades [52].

## Results

### Phylogenetic inference

**Figure 2:**
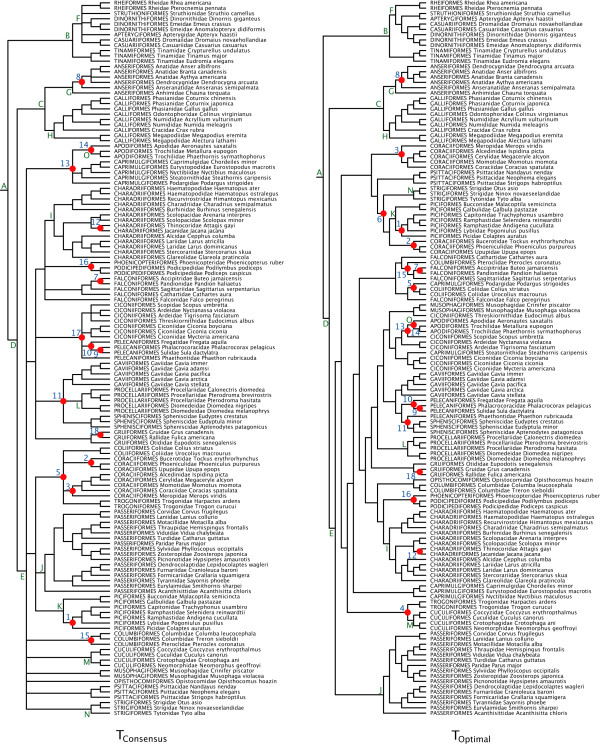
**Alternative tree topologies**. *T*_Consensus _(left) represents a conservative consensus estimate of avian familial relationships [53] (AIC_c _= 421460.9166). *T*_Optimal _(right) is our optimal topology derived from a partitioned model maximum likelihood search in RAxML (AIC_c _= 414160.2536). Some topological constraints were implemented in this search (see additional file 1). Solid circles and numbers indicate the placement of calibration points (see Table 5 for ages). Letters denote nodes whose age estimates are provided in Table 3.

Our optimal phylogenetic reconstruction (*T*_Optimal_; AIC_c _= 414160.2536) is a significantly better fit to the mtDNA matrix than a recent consensus topology derived from the literature (*T*_Consensus_; AIC_c _= 421460.9166; see the methods section and Figure 2). Nevertheless, the two topologies agree in many instances. For example, several traditional orders identified as having little support for monophyly (e.g. Coraciiformes, Ciconiiformes, Caprimulgiformes and Falconiformes [53]) were also polyphyletic in our analyses. However, the two trees also differ in many respects, most notably in the placement of Passeriformes. In *T*_Consensus_, the clade is relatively derived in the tree, whereas in *T*_Optimal _it forms the basal-most clade in Neoaves. Several traditionally hard-to-classify lineages (e.g. Pteroclidae, Opisthocomidae, Phaethontidae, Podargidae and Steatornithidae) are of suspect placement in *T*_Optimal_. These and other uncertainties tend to be localized and do not (as we report below) overly influence date estimates for the basal nodes in the avian tree. Some of the topological differences, however, are of operational importance, as they cause either redundancy or obsolescence of some fossil constraints used in estimating divergence times. Overall, of the 18 total internal fossil calibrations considered, 16 were used on *T*_Consensus_, and 17 on *T*_Optimal _(Figure 2).

### Divergence time estimation

A substantial signal was present for both a departure from a molecular clock and a lack of ancestor-descendant autocorrelation of substitution rates. Using penalized likelihood in r8s, both topologies *T*_Consensus _and *T*_Optimal _were found to be unclock-like, with optimal smoothing values (log_10λ_) of 1.0 and 0.4, respectively. Analyses in Dating5 clearly rejected the constant-rate Poisson model (-ln *L *= 63214.8; χ^2 ^= 8051.61, df = 271, *p *= 0.000) but could not reject the stationary (high variance) rate model (-ln *L *= 2123.01; χ^2 ^= 268.352, df = 269, *p *= 0.482) which produced a large index of dispersion *R *= 469.782. Bayesian analyses in Multidivtime delivered positive but very small values for the degree of autocorrelation of substitution rates across both topologies (Table 1). Finally, analyses of *T*_Consensus _using BEAST indicated that non-autocorrelated models of rate variation fit the data significantly better than a molecular clock (Table 2). Of the non-autocorrelated models, the lognormal distribution (UCLN) had a much better harmonic mean model likelihood than the exponential distribution (UCED), and relaxation (*T*_Flexible_) of a fixed topology further increased fit. Using each of these uncorrelated models, the covariance of substitution rates between ancestor and descendent branches across the tree was not significantly different from zero.

**Table 1:** Degree of autocorrelation in rates of molecular evolution by partition and tree topology as calculated in Multidivtime

Topology	Genetic partition	Autocorrelation (95% CI)
*T*_Consensus_	First positions	0.00197 (0.00127, 0.00290)
	Second positions	0.00437 (0.00258, 0.00685)
	Third positions	0.00452 (0.00288, 0.00680)
	RNA	0.00566 (0.00343, 0.00874)
*T*_Optimal_	First positions	0.00177 (0.00112, 0.00263)
	Second positions	0.00344 (0.00197, 0.00548)
	Third positions	0.00380 (0.00241, 0.00571)
	RNA	0.00414 (0.00206, 0.00744)

**Table 2:** Model comparisons for analyses relaxing the assumption of autocorrelation of rates across the tree. Harmonic mean model likelihoods were calculated from post-burnin MCMC samples generated in BEAST. For these model comparisons, the topology was fixed as *T*_Consensus_. The strict clock model serves as a base comparison. The tree *T*_Flexible _refers to analyses where topology is not fixed. Covariance indicates the degree of substitution rate autocorrelation between ancestor and descendent branches. 95% HPDs are given in parentheses.

Model	Model likelihood	Covariance
*T*_Consensus_		
CLOCK	-212231	N/A
UCED	-210459	0.039 (-0.103, 0.175)
UCLN	-207226	0.066 (-0.061, 0.193)
*T*_Flexible_		
UCLN	-206812	0.042 (-0.071, 0.161)

Given the extensive phylogenetic uncertainty within Neornithes, we focus on divergence times of clades for which monophyly is not contentious (Table 3). Two key trends are recognized. First, for a given dating method, differences in topology tended to have a minor but systematic influence on inferred ages. In general, *T*_Optimal _delivered older average date estimates than *T*_Consensus _using r8s (8.9 MY) and Multidivtime (3.6 MY), but the opposite trend was found with PATHd8 (-8.2 MY). When confidence/credibility intervals are considered, however, topology did not significantly influence most individual date estimates. Overall, in terms of the degree of discrepancy between fossil and molecular dates on a whole-tree basis (average δ_*Realized MA-FA*_), topology had a noticeable (> 5 MY) influence on divergence estimates for only the PATHd8 analyses (Table 3).

**Table 3:** Estimated divergence times for major avian clades compared across methods and topologies. Estimated time to most recent common ancestor (tMRCA) of clades of non-controversial monophyletic status. Standard deviations are given in parentheses (for Dating5 and BEAST, standard deviations were calculated from 95% confidence/credibility intervals using a normal approximation). For r8s, PATHd8 and Multidivtime ages were estimated on each of the two fixed topologies (*T*_Consensus _and *T*_Optimal_). For BEAST, divergence times were estimated simultaneously with phylogeny (*T*_Flexible_). For each method an estimate of the magnitude of disagreement between fossil and molecular estimates of divergence times (δ_*Realized MA-FA*_) is calculated as an average of MA-FA (the molecular age minus the fossil age) for those 18 internal nodes with calibration points.

		r8s		PATHd8		Multidivtime		Dating5	BEAST
Node	tMRCA	*T*_Consensus_	*T*_Optimal_	*T*_Consensus_	*T*_Optimal_	*T*_Consensus_	*T*_Optimal_	*T*_Optimal_	*T*_Flexible_

A	Neognaths-Paleognaths	125.7 (12.4)	131.1 (10.7)	114.3 (6.9)	102.8 (6.1)	129.9 (11.0)	132.4 (10.7)	132.9 (11.6)	133.2 (8.1)
B	Paleognaths	98.1 (10.6)	104.8 (10.7)	72.8 (5.0)	66.3 (4.6)	107.6 (11.2)	110.1 (11.2)	80 (6.8)	105.9 (11.7)
C	Galloanserae	93.6 (10.7)	100.7 (10.1)	86.4 (5.5)	78.7 (4.7)	97.3 (9.9)	100.6 (9.5)	89.3 (3.2)	110.4 (7.8)
D	Galloanserae-Neoaves	114.6 (12.1)	121.9 (10.5)	103.1 (6.0)	93.1 (5.4)	116.1 (11.0)	120.8 (10.5)	126.8 (6.1)	126.0 (7.1)
E	Neoaves	104.5 (11.4)	116.6 (9.9)	90.4 (5.1)	86.1 (5.0)	101.3 (10.1)	113.4 (10.1)	123.9 (5.3)	118.5 (6.8)
F	Ratites	67.4 (9.6)	89.3 (12.1)	49.5 (3.5)	46.7 (3.2)	92.1 (10.3)	97.3 (10.4)	40.6 (12.3)	91.5 (12.0)
G	Galliformes	82.1 (9.7)	88.4 (9.4)	82.2 (6.0)	73.2 (5.4)	87.4 (9.5)	87.2 (9.2)	67.3 (11.3)	99.0 (8.4)
H	Anseriformes	82.7 (10.1)	89.1 (10.7)	70.6 (4.0)	67.1 (2.8)	88.5 (9.3)	91.5 (9.0)	85.4 (4.1)	100.5. (8.3)
I	Charadriiformes	81.8 (11.5)	94.0 (9.2)	55.4 (3.6)	49.9 (3.1)	80.2 (8.5)	80.6 (7.8)	41.9 (4.5)	81.7 (6.3)
J	Passeriformes	85.4 (9.1)	99.5 (8.2)	89.0 (5.5)	85.5 (5.2)	78.4 (8.5)	97.8 (9.3)	84.9 (12.5)	106.6 (7.2)
K	Piciformes	90.9 (10.1)	99.6 (9.0)	89.0 (5.5)	79.0 (4.8)	83.0 (9.2)	91.1 (9.1)	101.0 (8.8)	93.6 (6.8)
L	Procellariiformes	73.8 (10.8)	89.9 (9.1)	55.6 (2.2)	38.1 (2.8)	80.0 (8.7)	78.5 (7.8)	38.8 (12.4)	74.7 (7.3)
M	Cuculiformes	73.9 (8.3)	79.5 (7.4)	65.0 (4.4)	60.1 (4.1)	68.3 (8.0)	74.3 (8.0)	52.5 (6.7)	74.1 (8.6)
N	Strigiformes	88.2 (9.9)	94.7 (8.6)	89.0 (5.9)	79.0 (5.1)	82.5 (9.7)	88.5 (9.4)	93.2 (11.5)	84.2 (9.1)
O	Apodiformes	77.4 (9.3)	75.1 (7.4)	70.0 (5.6)	53.5 (1.9)	77.3 (9.0)	63.4 (7.2)	55.8 (9.1)	80.5 (9.9)

	δ_*Realized MA-FA*_	39.8	44.6	24.2	16.9	36.8	36.1	24.8	36.5

Second, the choice of the relaxed clock method had a strong influence on inferred ages. R8s, Multidivtime and BEAST tended to deliver similar estimates for most clades of interest (Table 3). In contrast, PATHd8 generated considerably younger dates with much smaller confidence intervals, despite using the same bootstrapped phylograms and fossil constraints as r8s. Dating5 tended to produce the most extreme results, with inferred basal split estimates similar to those from Multidivtime, but some derived split estimates younger than those from PATHd8. Most significantly, PATHd8 and Dating5 together identified five of these major clades as having crown diversification restricted to the Cenozoic (Ratites, Charadriiformes, Procellariiformes, Cuculiformes and Apodiformes), although the remaining methods generate estimates for these same nodes that are on average more than 50% older. In terms of comparing molecular and fossil age estimates (average δ_*Realized MA-FA*_), r8s, Multidivtime and BEAST all show considerable discordance between the two sources of data, with the average molecular estimates for the major nodes (Table 3) being 36–45 MY older than corresponding fossil ages. PATHd8 and Dating5, in contrast, exhibit greater agreement between estimates from 'rocks' and 'clocks', with an average discrepancy of 17–25 MY.

**Figure 3:**
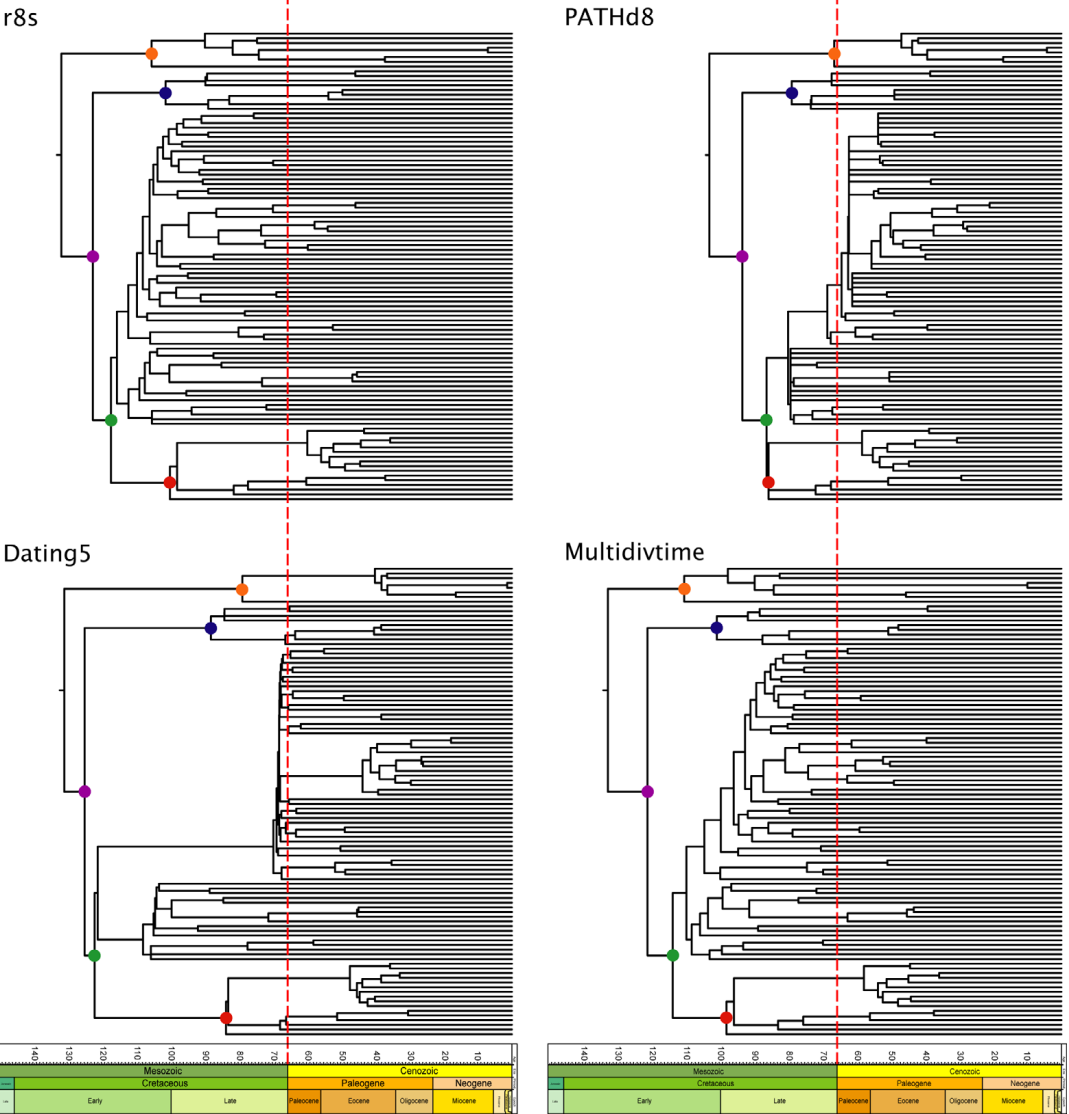
**Comparative timing of divergences for avian orders and families based on four different 'relaxed clock' methods**. Chronograms based on the optimal mtDNA tree reconstruction (*T*_Optimal_) using r8s (top left), Dating5 (bottom left), PATHd8 (top right) and Multidivtime (bottom right); see methods for explanation of differences between analytical approaches. For legibility, error bars are removed and trees are pruned to the family level. Filled circles denote major clades: orange, Paleognathae; purple, Neognathae; blue, Galloanserae; green, Neoaves; red, Passeriformes. Time is given in millions of years before present. The vertical dashed lines indicate the K-Pg boundary. r8s, Dating5 and Multidivtime reconstructions support Cretaceous origin and diversification. PATHd8 alone supports Cretaceous origin but Tertiary diversification.

**Figure 4:**
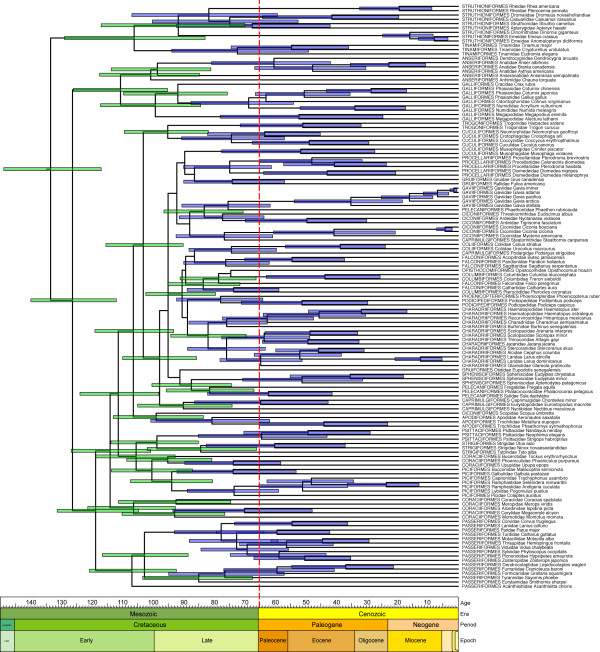
**A timeline for early avian evolution**. Maximum clade credibility (MCC) chronogram inferred using the non-autocorrelated model of rate evolution in BEAST while allowing topology to vary (*T*_Flexible_). Time is given in millions of years before present. The vertical dashed line indicates the K-Pg boundary. Error bars (blue and green) represent 95% posterior credibility intervals and are only given for nodes that were present on more than 50% of the posterior sampled trees. An unambiguous ancient diversification is indicated by 37 credibility intervals restricted to the Cretaceous (green bars).

**Figure 5:**
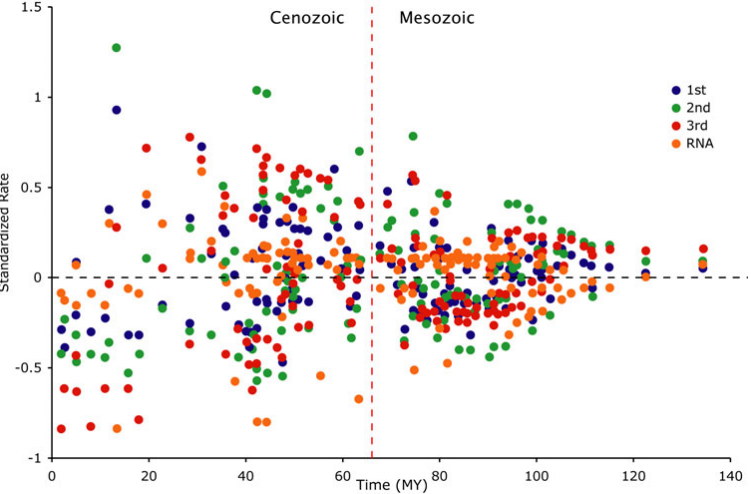
**Estimated rates of molecular evolution over time, in assessment of possible episodic evolution**. Standardized inferred rate of sequence evolution (per data partition) is plotted against inferred age for internal nodes on the optimal mtDNA tree reconstruction (*T*_Optimal_) using Multidivtime. Time is given in millions of years before present. No support is shown for an accelerated rate accompanying initial avian diversification or following the K-Pg boundary (vertical dashed line).

Despite these differences, all methods agree that the basal splits within Neornithes occurred deep within the Cretaceous (Table 3, nodes A-E). The youngest estimate for the root of Neornithes (PATHd8, *T*_Optimal_) is of Early Cretaceous age, 37 MY older than the oldest undisputed neornithean fossil [10]. Conflict among methods instead involves the diversification of derived lineages (Figures 3 and 4). Two patterns can be discerned. First, PATHd8 and Dating5 support bursts of speciation (many lineages arising almost simultaneously), whereas the remaining methods generally support more gradual diversification. Second, and more germane to the 'rock-clock' problem, PATHd8 alone supports an extensive post-K-Pg radiation of Neoaves. For example, from results of the non-autocorrelated rate models in BEAST allowing topological uncertainty (*T*_Flexible_; see Figure 4), not only are the basal splits inferred to have occurred in the Cretaceous, but 37 credibility intervals (green bars) do not overlap the K-Pg boundary. Finally, no support is shown for episodic evolution, either correlated with speciation events ([52]; no effect) or an increase in inferred substitution rate either during early diversification or following the K-Pg mass extinction (Figure 5).

## Discussion

### Phylogenetic inference

Whether using fossil or molecular data, a daunting impediment to divergence time estimation in birds is that resolution of many inter-ordinal phylogenetic relationships has proven obstinate, despite large data matrices with multiple character types [53]. Although our reconstruction *T*_Optimal _is overly optimistic in being fully resolved, it provides a useful alternative to the conservative *T*_Consensus _(Figure 2).

*T*_Optimal _recovers several traditional orders as polyphyletic (Caprimulgiformes, Coraciiformes, Falconiformes, Ciconiiformes), consistent with expectations [53] (but see [54]). Although *T*_Optimal _has caprimulgiform (nightbirds) families much more distantly related to one another than previous morphological [55] and molecular [56] investigations, differences in taxon sampling confounds direct comparison across studies. While Coraciiformes (kingfishers and relatives) is not found to be monophyletic, the two recovered sub-groupings both fall within a larger clade containing owls (Strigiformes), parrots (Psittaciformes) and woodpeckers and relatives (Piciformes). The monophyletic status of the order Falconiformes has received mixed support in previous analyses [54,57-61]. Placement of Falconidae in *T*_Optimal _is suspect and likely stems from insufficient taxon sampling from this family [62]. Regardless, no support was found for hypotheses uniting falconiform taxa with owls (Strigiformes) [54] or New World vultures (Cathartidae) with storks (Ciconiiformes) [61].

Several monotypic avian families have traditionally proved difficult to classify. The enigmatic hoatzin (Opisthocomidae) has variously been allied with at least four distantly related orders (Galliformes, Cuculiformes, Musophagiformes and Columbiformes; see [61,63]). We find here an alliance with doves (Columbiformes), similar to joint analyses of mitochondrial and nuclear DNA sequences [63]. The taxonomically problematic sandgrouse (family Pteroclidae) has alternatively been considered a member of Charadriiformes (shorebirds and allies [61,64]) or Columbiformes [54,57,60]. Our reconstruction has the sandgrouse distantly related to both orders, and instead allied with Falconiformes. This relationship is unsupported elsewhere and we have little confidence in this placement. The novel placement of the tropicbird (family Phaethontidae) as sister to Sphenisciformes is similarly suspect.

Finally, we find no support in our mtDNA analyses for the neoavian clades 'Metaves' and 'Coronaves' identified from nuclear β-fibrinogen intron analyses [58], although our constraint tree allowed for this possibility (*T*_Constraint_; see additional file 1). A major difference between these trees involves the phylogenetic position of the perching birds (Passeriformes); while nuclear DNA analyses recover Passeriformes as a relatively derived clade within 'Coronaves' [57,58], in *T*_Optimal _they instead comprise the basal-most lineage of Neoaves. This may be indicative of different phylogenetic signals in nuclear versus mtDNA sequences, as other mtDNA studies have also inferred a basal phylogenetic position of Passeriformes in Neoaves [65].

### Uncertainty in tree topology and substitution rate evolution

While *T*_Optimal _yields interesting hypotheses about avian relationships, the focus of this study is on estimating basal divergence times in Neornithes and we might regard topology as a nuisance parameter (and explicitly so in the BEAST *T*_Flexible _analyses). Topological error is usually not considered in divergence time estimation, but potentially could systematically bias age estimates through: (i) incorrect placement of fossil calibrations; and (ii) improper estimation of branch lengths. Through our joint consideration of *T*_Consensus _and *T*_Optimal_, we find that topology does have a systematic influence on inferred divergence times for nodes of interest (Table 3), but that for the present data set this influence differed in direction across methods and was generally insignificant when confidence/credibility intervals were considered. Removal of the restriction of a fixed topology in BEAST (*T*_Flexible_) yielded age estimates similar to those from Multidivtime analyses assuming *T*_Optimal_. Although yielding diffuse estimates, this 'relaxed topology' approach better reflects uncertainty in the underlying data.

An interesting result reported here is that rates of molecular evolution are found to be non-autocorrelated across the Neornithes tree (Tables 1 and 2), a result previously noted for virus and marsupial data sets [47]. An autocorrelation of rates would imply an inheritance of the trait 'rate of evolution'. This makes intuitive sense when considering that ancestor and descendant lineages are likely similar in body size, generation time, DNA repair efficiency, population size and other traits influencing rates of sequence evolution, and the most popular molecular dating methods available indeed implicitly assume that rates are autocorrelated across a tree [66,67]. However, even if 'rate of evolution' is heritable, nodes separated by long periods of time may accumulate sufficient rate variation that autocorrelation in this trait will break down. Alternatively, 'rate of evolution' may simply be more labile than we expect. Regardless, violation of an implicit autocorrelation assumption did not have significant effects on inferred dates for nodes of interest (Table 3). For example, r8s and Multidivtime, which each deal with rate variation in an ancestor-descendant fashion, deliver age estimates that match quite closely to those generated by BEAST, which does not make such an assumption.

### Antiquity of basal avian lineages

All methods employed here agree that the basal divergences within Neornithes occurred in the Cretaceous (Table 3, nodes A-E), supporting the refutation of a Cenozoic origin of modern lineages [8,9] mandated by the discovery of the 66 MY duck *Vegavis iaai *[10], which minimally forces five basal divergences into the Cretaceous. Moreover, our results are not dependent on this oldest fossil calibration, as analyses in r8s, PATHd8 and Multidivtime without using the *Vegavis *constraint returned nearly identical results to those reported here (data not shown); indeed, we must paradoxically conclude that this oldest undisputed neornithean fossil was essentially uninformative in our molecular dating analyses. Given the consensus across 'relaxed clock' methods employing very different assumptions about how molecular substitution rate evolves, we regard an Early Cretaceous origin of Neornithes as robustly supported. This inferred Cretaceous origin, and consequent survival of several avian lineages across the K-Pg boundary [68], is consistent with previous molecular studies [4,16-26] and is supported by historical biogeography reconstructions [69].

An explanation occasionally offered for the antiquity of molecular dates is that rates of sequence change may speed up during radiations [33], consistent with a basic tenet of punctuated equilibrium theory where most character change is concomitant with speciation [70], possibly resulting from stochastic effects during founder effect speciation [52,71]. If true, an elevated number of molecular substitutions recorded during diversification could erroneously be interpreted as a longer time span at a slower background rate, resulting in a systematic overestimation of divergence times for all nodes predating the radiation. Some evidence exists for a correlation between speciation and character evolution [52,72-74], although a study of island radiations failed to detect such an effect [75]. While punctuated molecular evolution may be less frequent in animals (18% of cases versus 44% and 60% in plants and fungi, respectively [52]), this effect is nevertheless a prime candidate to explain the strong and persistent discrepancy between molecular- and fossil-based divergence estimates. However, we find no signal for punctuated (speciational) molecular evolution [52] in the present data set. In addition, we fail to detect an accelerated rate associated with either the K-Pg boundary or during the initial diversification of Neornithes (Figure 5). If rates of sequence change were strongly influenced by diversification, we would expect clear departures from the inferred mean standardized substitution rate [76]. Although Cenozoic rates tend to be more variable than Mesozoic (ancestral) rates, we find no evidence for an obvious acceleration in substitution rate associated with any of the major nodes for any genetic partition. Although these two approaches to identifying episodic evolution would ideally involve more comprehensive taxon sampling, if punctuated evolution were primarily responsible for inflating inferred molecular dates then we likely would have detected the effect with the present data matrix.

Rather than narrowing the formidable 'rock-clock' gap through application of methods designed to minimize biases and accommodate uncertainty, we have instead considerably reinforced it. In part, the discordant age estimates can be explained through reference to the genuine time-lag (δ_*True MA-FA*_; see Figure 1) between the divergence of genetic lineages (predating speciation) and the evolution of diagnostic morphological characters (postdating speciation). However, given the estimated magnitude of δ_*Realized MA-FA *_(Table 3), it is unlikely that δ_*True MA-FA *_alone explains the dissonance. One the one hand, while the fossil record may be adequate for many evolutionary questions [28], there are clear instances where it is not. The 66 MY *Vegavis iaai *[10], for example, requires the coexistence of Paleognathae representatives; however, Cretaceous paleognaths are unknown. This may be a result of a geographical bias in fossil sampling favouring the northern hemisphere [2,17,69,77-79], as many taxa are hypothesized as having southern hemisphere (Gondwana) origins [69]. Although fossil gap analysis implies that a Cretaceous origin of several avian orders is unlikely [11], this method improperly assumes that fossils are randomly distributed (uniformly recovered through time) since the origin of a taxon; alternative fossil recovery curves can support very different scenarios, including scenarios that are more consistent with molecular genetic timelines [80], even when the fossil record is particularly sparse [31]. Although rightly considered with caution, the increasing number of fragmentary remains of putative neornithean lineages from the Cretaceous [78] lends credence to the ancient origin and diversification of Neornithes. On the other hand, there may yet be some unrecognized biases in the analysis of molecular genetic sequences, and our results suggest new directions for future avian divergence time studies (described below).

### Radiation of Neornithes

Despite broad agreement on the antiquity of basal divergences in Neornithes, arbitration among macroevolutionary models [1-4] to best describe the history of more derived lineages is complicated by disparity amongst various 'relaxed clock' results. Two important points of distinction can be recognized (Figures 3 and 4). First, Dating5 (overdispersed clock) and PATHd8 (rate smoothing across sister lineages) both infer bursts of speciation across the avian tree, while r8s (smoothing in an ancestor-descendant fashion), Multidivtime (Bayesian modelling of ancestor-descendant autocorrelated rate evolution) and BEAST (Bayesian modelling of rate evolution without an autocorrelation assumption or fixed topology) instead infer a more gradual diversification of Neornithes. Second, PATHd8 alone supports a prominent radiation of avian families in the Cenozoic, a scenario statistically rejected in many instances by the remaining four analyses. Although published PATHd8 divergence time estimates for Neoaves using nuclear DNA produced similarly young estimates [57], a reanalysis of these same data using Multidivtime roundly refuted the findings [16], echoing the incongruence of PATHd8 reported here. While the better reconciliation between molecular and fossil age estimates that PATHd8 offers seems satisfying at first, the unique discrepancy of this method from the other much more rigorous and biologically realistic methods raises concern.

Given the apparent lack of autocorrelation of substitution rates, together with the intrinsic topological uncertainty in the Neornithes tree, the analyses in BEAST best reflect our current understanding of early avian evolution (Figure 4). Without the restrictive assumptions inherent in other 'relaxed clock' methods, these analyses unambiguously support a Cretaceous origin and diversification of basal avian lineages. Even when considering large inferred credibility intervals, 37 early avian divergences are restricted to the Cretaceous, similar to that found through the analysis of nuclear DNA sequences [16]. It should be noted, however, that these nodes mostly represent order- and superfamily-level divergences; the majority of families sampled here (80 of 100 in BEAST) have their origins either overlapping or restricted to the Paleogene, consistent with interpretations from the fossil record [77]. In this respect, our results are similar to those inferred from a comprehensive study of the tempo of early mammalian evolution [81]. The results of both studies indicate that significant cladogenesis occurred in the Cretaceous, but that many crown groups diversified in the Cenozoic.

### Future progress

**Figure 6:**
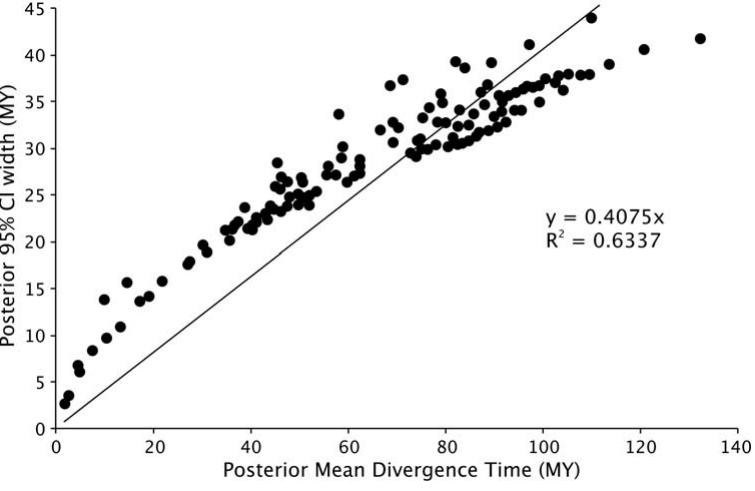
**Information content**. Posterior 95% credibility interval width is plotted against posterior mean divergence time using the results from Multidivtime on *T*_Optimal_. Here *R*^2 ^indicates the amount of information present in the data matrix and the regression coefficient is an estimate of the expected uncertainty that is independent of sequence length.

While there is a growing consensus for the Cretaceous origin of many Neornithes orders and families, the rate and timing of their diversification (and the influence of the K-Pg mass extinctions upon that diversification) is not yet resolved. MtDNA may have further utility in making progress on the problem, as our analysis of posterior credibility interval widths indicates that longer sequences would likely improve divergence time estimates (Figure 6). However, we recognize that mtDNA is limited in that all sites belong to a single 'super-locus', and so a significant reduction of uncertainty (e.g. the slope in Figure 6) will ultimately require the supplement of multiple independent nuclear loci. In addition, while the present study was focused at the family level, increased taxon sampling will break up long branches (benefiting phylogenetic reconstruction) and improve branch length estimates. Nevertheless, our results suggest fertile ground for future molecular research into this problem. For example, we find that: (i) variation in the number of substitutions across branches can be accommodated by a high variance stationary-rate model [82]; and (ii) rates are not autocorrelated across the avian tree in an ancestor-descendent fashion. This suggests potential for development of a hybrid model that accommodates both pieces of information and that future studies test assumptions of rate autocorrelation before invoking them, at least for the tree depth that we consider here.

In regards to the 'rock-clock' debate [33,49], we feel that much of the perceived dissonance between fossil- and molecular-based age estimates stems from a comparison of different phylogenetic entities: molecular phylogeneticists generally deal with stem-group origins, while paleontologists generally concentrate on crown-group diversification [4]. Moreover, it is too rarely emphasized that when dating the same node a genuine discrepancy is expected, as coalescent times (*T*_gene_; see Figure 1) will predate cladogenesis while morphological diversification (*T*_morphology_; see Figure 1) will postdate cladogenesis. The reality is that in normal practice neither group directly addresses the main parameter of interest, the timing of speciation (*T*_species_; see Figure 1), the pattern of which is essential to the understanding of the origins of biodiversity. However, tools do exist to better estimate speciation times from both fossils (e.g. accounting for 'ghost' lineages [31]) and genetic data (e.g. explicitly modelling ancestral effective population sizes to account for coalescent times [83]). Further, molecular methods can be augmented with greater information from the fossil record, possibly by incorporating models of preservation bias into temporal constraints [31]. Newly developed methods exist where probability distributions can be applied to calibrated nodes in a Bayesian framework [47,84,85]. Although we recognize that this approach is superior in that it can lend more credence to the fossil record than standard minimum-age constraints, we refrained from using such methods here as there is currently insufficient information with which to specify meaningful prior distributions for most avian diversification times. Realization of such distributions will require more communication between paleontologists and molecular phylogeneticists [86,87].

## Conclusion

One possible explanation for the discrepancy between molecular and fossil data in dating early divergences of avian lineages has been that the genetic data have been misinterpreted. In this vein, the ancient age estimates reported from previous molecular studies are seen as artefacts of oversimplified or improperly executed methods. Here we have examined this conjecture by accommodating uncertainty in genetic divergence time estimates. Through analyses of a large mtDNA matrix using multiple cladistically tested calibrations, alterative tree topologies and several sophisticated relaxed clock methods we have found that the molecular estimates are robust to varying assumptions about the evolution of evolutionary rates and consistent with those from previous studies. Our findings thus strongly support pre-K-Pg genetic origins for multiple modern bird lineages, including various extant orders and families, in contrast to the model of post-K-Pg diversification derived from a narrow interpretation of the fossil record.

## Methods

### Molecular sequence data

The molecular data set comprises 135 avian species and 100 traditionally recognized families, as well as five outgroup taxa (turtles, *n *= 3; crocodilians, *n *= 2; see Additional file 2). The names for avian taxa shown in our figures and tables generally follow Gill and Wright [88]. Turtles were used solely to root phylogenies and were not used for dating purposes. For each taxon a total of 5248 base pairs (bp) of mitochondrial DNA (mtDNA) was either sequenced directly using primers reported by or modified from [89] or downloaded from GenBank. mtDNAs from taxa newly collected by us and first reported here (GenBank accession numbers EU166921–EU167086, EU372666–EU372688, EU391159). Protein-coding genes were aligned at the amino acid level, while RNA genes were aligned as nucleotides using secondary structure models following [90]. From the original matrix, 654 alignment positions could not be aligned unambiguously and so were excluded from subsequent analyses, yielding a final matrix of 4594 bp (Table 4).

**Table 4:** Aligned fragment lengths of mtDNA sequences (total 4594 bp). Codon positions are combined across all protein-coding genes (ND1, ND2, cytochrome *b*), and RNA includes 12S and nine tRNA genes (L, I, Q, M, W, A, N, C, Y).

Gene	Aligned length (bp)	Variable sites (%)	Parsimony informative sites
First codon positions	1043	699 (67%)	605
Second codon positions	1043	512 (49%)	380
Third codon positions	1043	1043 (100%)	1041
RNA	1465	910 (62%)	728

### Fossil calibration points

We include as many fossil calibration points as possible in disparate parts of the avian tree to maximize information from the fossil record, and reduce dependency on any one calibration estimate. Given that serious bias can result if even a single fossil has been misdiagnosed in its taxonomic affinity, we limit our calibration points to those fossils that have been rigorously analyzed in a cladistic context (Table 5). Our fossil calibrations are nearly identical to those used by Brown et al [16], which is a modification of the fossil complement used by Ericson et al [57]. These internal avian calibration points are all implemented as minimum age constraints in the various dating analyses. We also use two bounded external calibrations derived from the fossil record that date the caiman-alligator (71-66 MY) and crocodile-bird (251-243 MY) splits [91]. This last calibration has recently been independently supported by molecular data using soft calibration bounds [92].

**Table 5:** Fossil calibrations. All internal calibrations for Neornithes are treated as minimum ages. External calibrations are treated as bounded (lower, upper) constraints. See [57] for fossil citations and details. See Figure 2 for placement of calibrations in the alternative trees.

Fossil ID	Calibration	Age (MY)	Source
1	Crown Pici	30	[57]
2	Stem Upupidae + Phoeniculidae	47.5	[57]
3	Stem Coraciidae + Brachypteraciidae	47.5	[57]
4	Stem Trogoniformes	53	[57]
5	Stem Coliiformes	55	[57]
6	Stem Strigiformes	55	[57]
7	Crown Pandionidae	37	[57]
8	Stem Anatidae	66	[10]
9	Crown Sulidae	33	[57]
10	Stem Fregatidae	53	[57]
11	Stem Sphenisciformes	62	[24]
12	Stem Jacanidae	30	[57]
13	Stem Apodiformes	53	[57]
14	Stem Trochilidae	30	[109]
15	Crown Pteroclididae	30	[57]
16	Stem Phoenicopteriformes	30	[57]
17	Stem Phaethontidae	55	[57]
18	Stem Gruidae	30	[57]
20	Alligator-caiman	66–71	[91]
21	Bird-crocodile	243–251	[91]

### Phylogenetic trees and branch length uncertainty

Inferring dates on an incorrect tree topology will lead to estimates that are suspect, if not wholly incorrect. We seek to accommodate the existing uncertainty about avian phylogenetic relationships by dating nodes on two alternative trees. The first topology considered is taken from Figure 27.10 of Cracraft et al [53], which is a consensus tree based on previous molecular- and morphology-based phylogenetic reconstructions. This tree is conservative in that all represented branching events are well supported by independent lines of evidence; uncertain relationships among lineages are presented as hard polytomies. This topology is referred to as *T*_Consensus_. The second topology considered was derived from a partitioned-model maximum likelihood search using the program RAxML-VI-HPC 2.2.3 [93]. The data were divided into four partitions: first, second and third codon positions of mitochondrial cytochrome-b, ND1 and ND2 genes, and concatenated mitochondrial 12S rDNA and tRNA genes (L, I, Q, M, W, A, N, C, Y). A collapsed consensus tree derived from Cracraft et al (thick branches only of Figure 27.10 in [53]) was used as a backbone constraint to make tree searches more efficient (*T*_Constraint_; see Additional file 1). Several hundred heuristic searches were performed under the GTRMIX model assuming a range of values for both the initial rearrangement setting (*i*; range 5–20) and number of rate categories (*c*; range 5–25). The topology of the maximum likelihood estimate (MLE) is referred to as *T*_Optimal_.

For the non-Bayesian dating methods, we accommodate uncertainty in branch length estimation through a non-parametric bootstrapping procedure. For each original data partition, 100 pseudoreplicate datasets were generated using the program SEQBOOT of the PHYLIP 3.6 package [94]; these bootstrapped matrices were concatenated to produce 100 pseudomatrices with the same size and number of partition-specific characters as the original matrix. For *T*_Optimal_, branch lengths and substitution model parameters were estimated using a partitioned GTR+G model in RAxML. For *T*_Consensus_, branch lengths and all substitution model parameters were estimated from each bootstrap matrix on the fixed topology using the GTR+I+G substitution model in PAUP* [95] because RAxML cannot evaluate a tree containing mutlifurcations. Using these procedures we generated 100 trees with branch lengths (phylograms) for each topology.

### Divergence time estimates using relaxed molecular clocks

Owing to the concern that assumptions of particular analytical methods may systematically bias divergence time estimates, we compare several methods for accommodating among-lineage rate heterogeneity for the purpose of estimating the divergence times of modern avian lineages. To facilitate direct comparison, all methods utilize the same fossil calibrations and tree topologies outlined above.

First, the program r8s 1.7 [66] was used to estimate rates and dates for internal nodes in the phylogeny via penalized likelihood (PL). This semiparametric procedure combines a parametric model that allows for different rates on each branch in the tree [96] with a non-parametric penalty function that penalizes rates that change too quickly along the tree from ancestor to descendent branches [97]; a smoothing parameter (λ) determines the relative contribution of the penalty function. The optimal smoothing value was determined individually for each topology through a sequence-based cross-validation procedure [96] using penalty = add and the normalized (χ^2^) errors. Confirmation of the optimal smoothing values was obtained via multiple optimizations with different initial conditions (by setting num_restarts = num_time_guesses = 3). Confidence intervals for node ages were determined using the distribution of estimated ages across bootstrapped trees assuming the optimal smoothing value from the original phylograms. Summary of the mean estimate and associated error for a given node was accomplished using the profile command in r8s.

Second, the program PATHd8 [98,99] also smoothes rates across the tree, but does so by calculating an average path length from an internal node to all its descending terminals; deviations from a molecular clock are corrected through reference to fossil calibrations. Important distinctions from r8s above are that PATHd8 smoothes rates between sister groups (rather than from ancestor to descendent nodes) and it does this locally rather than over the entire tree. The same 100 phylograms as analyzed with r8s above were used to generate confidence intervals on divergence times, although summary statistics were calculated manually.

As a third approach, the Bayesian MULTIDISTRIBUTE package [100] represents an attempt to explicitly model rate change across a tree [67,101,102]. Here, the logarithm of the rate at the end of a branch is modelled with a normal distribution, the mean of which has an expected value equal to the rate at the beginning of the branch; thus, rates are implicitly assumed to be autocorrelated from ancestor to descendent nodes, although this autocorrelation may decay with increasing branch lengths. The posterior probability distribution of divergence times is approximated by samples from a Markov chain Monte Carlo (MCMC) chain. The data were partitioned as noted above. For each partition, estimates of the transition/transversion rate ratio and the gamma site class-specific rates under the F84+G model (the most complex model supported by the MULTIDISTRIBUTE package) were calculated in the baseml program of the PAML 3.15 package [103]. The output from baseml was used as the input for the MULTIDISTRIBUTE program estbranches, which produced MLEs of branch lengths and their approximate variance-covariance matrix. Although differing in branching order, *T*_Consensus _and *T*_Optimal _had similar overall tree lengths; as a result, the same priors were applied to both topologies in the program Multidivtime: rtrate = rtratesd = 0.012, and brownmean = brownmeansd = 0.01. As one of our external calibration points bounds the root node (crocodile-bird split at 251-243 MY), date priors were less important and were defined narrowly (bigtime = 255 MY, rttm = 247 MY, rttmsd = 1.5 MY). The program was run without the assumption of correlated changes in rates across data partitions. Following a burnin of 10^6 ^samples, 10^4 ^samples were taken at a sampling interval of 10^2^. All analyses were repeated with different inseed values to check for convergence of the MCMC chain.

Fourth, the overdispersed clock method of Cutler [82] represents a strong departure from other approaches to dating in the way it models branch length heterogeneity. Instead of treating a variable number of substitutions across lineages as indicative of changes in substitution rate across the tree, Cutler's method assumes that rate is stationary, but with high variance. Thus, rather than assuming that the number of substitutions along a lineage is Poisson distributed (where the variance is equal to the mean), the observed variation across branches is accommodated by the larger variance afforded through a Gaussian distribution. As a result, branches with either particularly high or low numbers of substitutions need not be clustered on the tree; in other words, heritability (phylogeny) plays no role in the observed numbers of substitutions. The program Dating5 [104] calculates χ^2 ^statistics for both the constant-rate Poisson and stationary models and compares the overall fit of the models using a likelihood ratio test. In addition, the program calculates *R*, the index of dispersion (the ratio of the variance to the mean number of substitutions) under the stationary model. Dating was restricted to *T*_Optimal _as the current version of Dating5 does not allow for polytomies. In addition, we could not achieve likelihood convergence with the partitioned data, and so reported results are from concatenated sequences. From asymptotic likelihood theory, 95% confidence intervals were calculated using a threshold of 2 log likelihood units around the MLE.

Finally, the program BEAST 1.4.6 [105] differs in two important ways from the dating methods listed above. First, it does not require a fixed topology; rather, branch lengths, topology, substitution model parameters and dates can be estimated simultaneously. This incorporation of uncertainty in topology may be particularly important for the present data set, where relationships amongst many clades are uncertain. Second, BEAST does not assume that substitution rates are necessarily autocorrelated across the tree. Although intuitively satisfying, autocorrelation of rates has not been demonstrated in the literature; in fact, Drummond et al [47] report that many empirical data sets do *not *exhibit significant autocorrelation of rates.

BEAST 1.4.6 offers two statistical distributions for describing the change in rate across a branch [47]: rates can be drawn independently from either an exponential distribution (UCED) or a lognormal distribution (UCLN). To find which distribution fit the present data best, we initially fixed the tree topology to *T*_Consensus_. The data were partitioned as above, with each partition assigned a GTR+I+G substitution model. BEAST MCMC runs of 25 × 10^6 ^generations following a burnin of 10^5 ^generations were performed for UCED, UCLN and CLOCK models. To arbitrate between models, we calculated Bayes factors by comparing harmonic mean model likelihoods [106]. For both non-autocorrelated models, we also calculated the covariance among branch rates, which indicates the degree of ancestor-descendant autocorrelation of rates across the tree. Using the optimal model, we then accommodated topological uncertainty by removing the restriction of a fixed tree. However, we did constrain certain clades (the backbone constraints described above) to be monophyletic to facilitate the placement of calibration points and increase the efficiency of the MCMC search. Three replicate runs of 25 × 10^6 ^generations were performed to check for convergence of the MCMC chain. Mean parameter estimates and 95% highest posterior densities (HPDs) were determined through analyzing the combined BEAST tree files in TreeAnnotator 1.4.6 [107]. We refer to these results with the topology *T*_Flexible_.

For each of the five dating methods above we calculated the average discrepancy between molecular (MA) and fossil (FA) estimated ages for those nodes that had fossil calibrations. The MA used in these calculations was the mean estimate from MCMC samples (Multidivtime, BEAST), or the optimal estimate from the empirical data matrix (r8s, PATHd8, Dating5). The value MA-FA is equivalent to δ_*Realized MA-FA *_described above, and gives an indication of the degree of agreement between the molecular data and the fossil record.

### Episodic evolution and information content

If present, episodic (or punctuated) molecular evolution could seriously bias molecular genetic estimates of divergence time by systematically overestimating the ages of all nodes that preceded the punctuation. We therefore tested for the presence of episodic evolution in two ways. First, we used the method of Pagel et al [52,108] which quantifies the proportional contribution of punctuated (β) and gradual (g) evolution to path lengths in a phylogeny, based on extent of association between sequence change and cladogenesis events. This method requires a fully bifurcating tree, and so analyses were limited to our optimal reconstruction *T*_Optimal_. To test for this signature we analyzed maximum likelihood trees from RAxML bootstrap analyses (*n *= 100). Second, to test whether substitution rate accelerated coincident with or following the K-Pg boundary we plotted standardized inferred substitution rate (per data partition) versus inferred divergence time from the Multidivtime analyses above. If the K-Pg boundary extinctions facilitated diversification of avian higher-level taxa, it could produce a spike in this plot [76] detected as a departure from the mean standardized rate. These two tests are complementary in that the first focuses specifically on the effects of speciation, whereas the second focuses on absolute time effects, which may or may not be related to speciation.

As in the case of episodic evolution, a simple lack of historical signal present in molecular sequences could generate erroneous divergence time estimates. We therefore assessed the 'information content' present in our mtDNA matrix by regressing posterior 95% credibility width against posterior mean divergence time. When the when the amount of data is saturated then this regression will produce a linear relationship (i.e. *R*^2 ^→ 1), the slope of which indicates the amount of uncertainty that cannot be reduced through adding longer sequences [7,85], but can be reduced through adding independent loci.

## Abbreviations

AIC_*c*_, corrected Akaike Information Criterion; bp, base pairs; CLOCK, strict molecular clock; CV, cross validation; FA, fossil age; HPD, highest posterior density; K-Pg, Cretaceous-Paleogene boundary; MA, molecular age; MCC, maximum clade credibility; MCMC, Markov chain Monte Carlo; MLE, maximum likelihood estimate; mtDNA, mitochondrial DNA; MY, millions years; PL, penalized likelihood; tMRCA, time to the most recent common ancestor; UCED, uncorrelated exponential distribution; UCLN, uncorrelated lognormal distribution.

## Authors' contributions

JWB conducted the reported phylogenetic reconstructions and all dating analyses, and drafted the manuscript. JSR performed the multiple sequence alignments and initial phylogenetic analyses. JG-M and MDS conducted most of the molecular sequencing. DPM designed and developed the study. All authors contributed to revisions yielding the final manuscript.

## Supplementary Material

Additional file 1 Supplemental figure S1 Constraint tree. A consensus tree derived from the thick branches only of Figure 27.10 of Cracraft et al. [53] used as a backbone constraint in RAxML tree searches. Click here for file

Additional file 2 Supplemental table S1 GenBank accession numbers. Accession number information for analyzed sequence data (sequences EU166921–EU167086, EU372666–EU372688, and EU391159 are novel to this study). Click here for file
