# Supplementary figures and images for: Strong mitochondrial DNA support for a Cretaceous origin of modern avian lineages
Source: BMC Biol. 2008 Jan 28;6:6. doi: 10.1186/1741-7007-6-6 (PMC2267772; doi:10.1186/1741-7007-6-6)

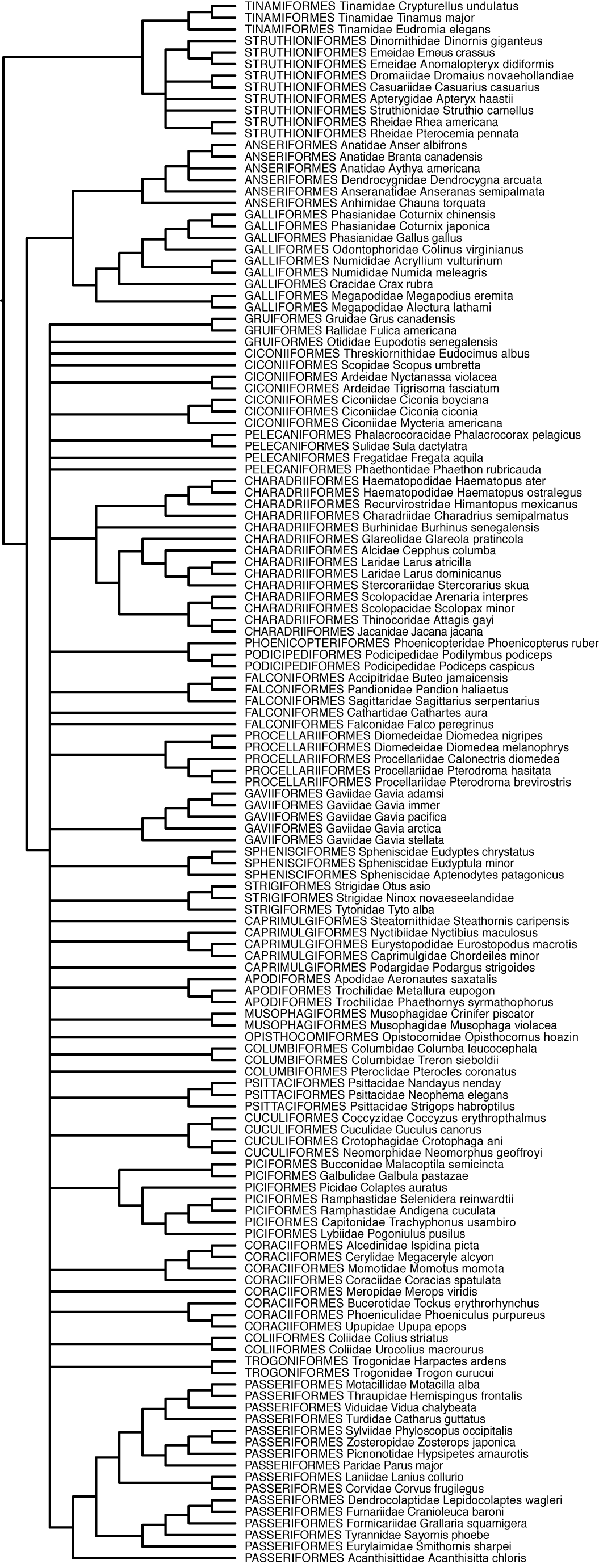

Supplement: Additional file 1 — Supplemental figure S1 Constraint tree. A consensus tree derived from the thick branches only of Figure 27.10 of Cracraft et al. [53] used as a backbone constraint in RAxML tree searches. [file 1741-7007-6-6-S1.png]
