# Supplementary material for: Strong mitochondrial DNA support for a Cretaceous origin of modern avian lineages
Source: BMC Biol. 2008 Jan 28;6:6. doi: 10.1186/1741-7007-6-6 (PMC2267772; doi:10.1186/1741-7007-6-6)
Supplement: Additional file 2 — Supplemental table S1 GenBank accession numbers. Accession number information for analyzed sequence data (sequences EU166921–EU167086, EU372666–EU372688, and EU391159 are novel to this study). [file 1741-7007-6-6-S2.doc]

# Additional files

Additional file 1 – Sample information for taxa used in this study.

| Order | Family | Species | Common Name | 12S | CYTB | ND1 | ND2 | tRNAs |
| --- | --- | --- | --- | --- | --- | --- | --- | --- |
| Testudines |  |  |  |  |  |  |  |  |
|  | Cheloniidae | Chelonia mydas | Green sea-turtle | AB012104 | AB012104 | AB012104 | AB012104 | AB012104 |
|  | Emydidae | *Chrysemys picta* | Painted turtle | NAF069423 | NAF069423 | NAF069423 | NAF069423 | NAF069423 |
|  | Pelomedusidae | *Pelomedusa subrufa* | African helmeted turtle | AF039066 | AF039066 | AF039066 | AF039066 | AF039066 |
| Crocodylia |  |  |  |  |  |  |  |  |
|  | Crocodylidae | Alligator mississipiensis | American alligator | Y13113 | Y13113 | Y13113 | Y13113 | Y13113 |
|  | Crocodylidae | *Caiman crocodilus* | Spectacled caiman | AJ404872 | AJ404872 | AJ404872 | AJ404872 | AJ404872 |
| **Paleognathae** | | | | | | | | |
| Struthioniformes | Apterygidae | *Apteryx haastii* | Great spotted kiwi | AF338708 | AF338708 | AF338708 | AF338708 | AF338708 |
| Struthioniformes | Casuariidae | *Casuarius casuarius* | Southern cassowary | AF338713 | AF338713 | AF338713 | AF338713 | AF338713 |
| Struthioniformes | Dromaiidae | *Dromaius novaehollandiae* | Emu | AF338711 | AF338711 | AF338711 | AF338711 | AF338711 |
| Struthioniformes | Dinornithidae | *Dinornis giganteus* | Giant moa | AY016013 | AY016013 | AY016013 | AY016013 | AY016013 |
| Struthioniformes | Emeidae | *Anomalopteryx didiformis* | Little bush moa | AF338714 | AF338714 | AF338714 | AF338714 | AF338714 |
| Struthioniformes | Emeidae | *Emeus crassus* | Eastern moa | AY016015 | AY016015 | AY016015 | AY016015 | AY016015 |
| Struthioniformes | Rheidae | *Pterocnemia pennata* | Lesser rhea | AF338709 | AF338709 | AF338709 | AF338709 | AF338709 |
| Struthioniformes | Rheidae | Rhea americana | Greater rhea | AF090339 | AF090339 | AF090339 | AF090339 | AF090339 |
| Struthioniformes | Struthionidae | *Struthio camellus* | Ostrich | AH007281 | AH007281, Y12025 | AH007281 | AH007281 | AF069429 |
| Tinamiformes | Tinamidae | *Crypturellus undulatus* | Undulated tinamou | AY139627 | AY139629 | AY139628 | AY139628 | AY139628 |
| Tinamiformes | Tinamidae | *Eudromia elegans* | Elegant crested tinamou | AY274002 | AY274002 | AY274002 | AY274002 | AY274049 |
| Tinamiformes | Tinamidae | *Tinamus major* | Great tinamou | AF338707 | AF338707 | AF338707 | AF338707 | AF338707 |
| **Galloanserae** | | | | | | | | |
| Anseriformes | Anatidae | *Anser albifrons* | Greater white-fronted goose | AF363031 | AF363031 | AF363031 | AF363031 | AF363031 |
| Anseriformes | Anatidae | *Aythya americana* | Redhead | AF069422 | AF069422 | AF069422 | AF069422 | AF090337 |
| Anseriformes | Anatidae | *Branta canadensis* | Canada goose | DQ019124 | DQ019124 | DQ019124 | DQ019124 | DQ019124 |
| Anseriformes | Anhimidae | *Chauna torquata* | Southern screamer | AY274030 | AY274006 | AY274053 | AY274053 | AY274053 |
| Anseriformes | Anseranatidae | *Anseranas semipalmata* | Magpie goose | AF173772 | U83730 | AY274054 | AY274054 | AY274054 |
| Anseriformes | Dendrocygnidae | *Dendrocygna arcuata* | Wandering whistling-duck | AF536743 | AF536740 | AF536746 | AF536746 | U97735 |
| Galliformes | Cracidae | *Crax rubra* | Great curassow | AY274029 | AY274003 | AY274050 | AY274050 | AY274050 |
| Galliformes | Megapodidae | *Megapodius eremita* | Melanesian megapode | AF082065 | AY274005 | AY274052 | AY274052 | AY274052 |
| Galliformes | Megapodidae | *Alectura lathami* | Brush turkey | AF082058 | AY274004 | AY274051 | AY274051 | AY346091 |
| Galliformes | Numididae | *Acryllium vulturinum* | Vulturine guineafowl | AF536742 | AF536739 | AF536745 | AF536745 | AF536745 |
| Galliformes | Numididae | *Numida meleagris* | Helmeted guineafowl | AP005595 | AP005595 | AP005595 | AP005595 | AP005595 |
| Galliformes | Odontophoridae | *Colinus virginianus* | Northern bobwhite | EU167061 | XXXXXX | EU166949 | EU166949 | EU166949 |
| Galliformes | Phasianidae | *Coturnix chinensis* | King quail | AB073301 | AB073301 | AB073301 | AB073301 | AB073301 |
| Galliformes | Phasianidae | *Coturnix japonica* | Japanese quail | AP003195 | AP003195 | AP003195 | AP003195 | AP003195 |
| Galliformes | Phasianidae | *Gallus gallus* | Red junglefowl | X52392 | X52392 | X52392 | X52392 | X52392 |
| **Neoaves** | | | | | | | | |
| Apodiformes | Apodidae | *Aeronautes saxatalis* | White-throated swift | EU167032 | EU166978 | EU166921 | EU166921 | EU166921 |
| Apodiformes | Trochilidae | *Metallura eupogon* | Fire-throated metaltail | EU167083 | EU167027 | EU166922 | EU166922 | EU166922 |
| Apodiformes | Trochilidae | *Phaethornis syrmathophorus* | Tawny-bellied hermit | EU167084 | EU167028 | EU166923 | EU166923 | EU166923 |
| Caprimulgiformes | Caprimulgidae | *Chordeiles minor* | Common nighthawk | EU167037 | EU166983 | EU166924 | EU166924 | EU166924 |
| Caprimulgiformes | Eurystopodidae | *Eurostopodus macrotis* | Great eared-nightjar | EU167043 | EU166989 | EU166925 | EU166925 | EU166925 |
| Caprimulgiformes | Nyctibidae | *Nyctibius maculosus* | Andean potoo | EU167060 | EU167006 | EU166926 | EU166926 | EU166926 |
| Caprimulgiformes | Podargidae | *Podargus strigoides* | Tawny frogmouth | EU167069 | EU167014 | EU166927 | EU166927 | EU166927 |
| Caprimulgiformes | Steatornithidae | *Steatornis caripensis* | Oilbird | EU167079 | EU167023 | EU166928 | EU166928 | EU166928 |
| Charadriiformes | Alcidae | *Cepphus columba* | Pigeon guillemot | XXXXXX | XXXXXX | XXXXXX | XXXXXX | XXXXXX |
| Charadriiformes | Burhinidae | *Burhinus senegalensis* | Senegal thick-knee | AY274043 | AY274007 | AY274073 | AY274073 | AY274073 |
| Charadriiformes | Charadriidae | *Charadrius semipalmatus* | Semipalmated plover | EU167040 | EU166986 | EU166929 | EU166929 | EU166929 |
| Charadriiformes | Glareolidae | *Glareola pratincola* | Collared pratincole | XXXXXX | XXXXXX | XXXXXX | XXXXXX | XXXXXX |
| Charadriiformes | Haematopodidae | *Haematopus ater* | Blackish oystercatcher | AY074886 | AY074886 | AY074886 | AY074886 | AY074886 |
| Charadriiformes | Haematopodidae | *Haematopus ostralegus* | Eurasian oystercatcher | EU167052 | EU166998 | EU166930 | EU166930 | EU166930 |
| Charadriiformes | Jacanidae | *Jacana jacana* | Wattled jacana | EU167053 | EU166999 | EU166935 | EU166935 | EU166935 |
| Charadriiformes | Laridae | *Larus atricilla* | Laughing gull | EU167055 | EU167001 | EU166931 | EU166931 | EU166931 |
| Charadriiformes | Laridae | *Larus dominicanus* | Kelp gull | AY293619 | AY293619 | AY293619 | AY293619 | AY293619 |
| Charadriiformes | Recurvirostridae | *Himantopus mexicanus* | Black-necked stilt | EU167077 | EU167022 | EU166932 | EU166932 | EU166932 |
| Charadriiformes | Scolopacidae | *Arenaria interpres* | Ruddy turnstone | AY074885 | AY074885 | AY074885 | AY074885 | AY074885 |
| Charadriiformes | Scolopacidae | *Scolopax minor* | American woodcock | AF082068 | U83744 | AY274072 | AY274072 | AY274072 |
| Charadriiformes | Stercorariidae | *Stercorarius skua* | Pomarine skua | EU167080 | EU167024 | EU166933 | EU166933 | EU166933 |
| Charadriiformes | Thinocoridae | *Attagis gayi* | Rufous-bellied seedsnipe | EU167081 | EU167025 | EU166934 | EU166934 | EU166934 |
| Ciconiiformes | Ardeidae | *Nyctanassa violacea* | Yellow-crowned night heron | EU167033 | EU166979 | EU166936 | EU166936 | EU166936 |
| Ciconiiformes | Ardeidae | *Tigrisoma fasciatum* | Fasciated tiger heron | EU167034 | EU166980 | EU166937 | EU166937 | EU166937 |
| Ciconiiformes | Ciconiidae | *Ciconia boyciana* | Oriental wood stork | AB026193 | AB026193 | AB026193 | AB026193 | AB026193 |
| Ciconiiformes | Ciconiidae | *Ciconia ciconia* | European wood stork | AB026818 | AB026818 | AB026818 | AB026818 | AB026818 |
| Ciconiiformes | Ciconiidae | *Mycteria americana* | Wood stork | AF082066 | U83712 | AY274076 | AY274076 | AY274076 |
| Ciconiiformes | Scopidae | *Scopus umbretta* | Hamerkop | XXXXXX | U08936 | XXXXXX | XXXXXX | XXXXXX |
| Ciconiiformes | Threskiornithidae | *Eudocimus albus* | White ibis | EU167082 | EU167026 | EU166938 | EU166938 | EU166938 |
| Coliiformes | Coliidae | *Colius striatus* | Spectacled mousebird | AY274032 | AY274011 | AY274058 | AY274058 | AY274058 |
| Coliiformes | Coliidae | *Urocolius macrourus* | Blue-naped mousebird | AY274033 | AY274012 | AY274059 | AY274059 | AY274059 |
| Columbiformes | Columbidae | *Columba leucocephala* | White-crowned pigeon | AY274041 | AY274023 | AY274070 | AY274070 | XXXXXX |
| Columbiformes | Columbidae | *Treron sieboldii* | White-bellied green-pigeon | AY274042 | AY274024 | AY274071 | AY274071 | XXXXXX |
| Columbiformes | Pteroclidae | *Pterocles coronatus* | Crowned sandgrouse | EU167073 | EU167018 | EU166939 | EU166939 | EU166939 |
| Coraciiformes | Alcedinidae | *Ispidina picta* | African kingfisher | EU167031 | EU166977 | EU166940 | EU166940 | EU166940 |
| Coraciiformes | Bucerotidae | *Tockus erythrorhynchus* | Red-billed hornbill | AF082071 | AY274008 | AY274055 | AY274055 | AY274055 |
| Coraciiformes | Cerylidae | *Megaceryle alcyon* | Belted kingfisher | EU167039 | EU166985 | EU166945 | EU166945 | EU166945 |
| Coraciiformes | Coraciidae | *Coracias spatulata* | Racquet-tailed roller | AF082060 | AY274010 | AY274057 | AY274057 | AY274057 |
| Coraciiformes | Meropidae | *Merops viridis* | Blue-throated bee-eater | EU167057 | EU167003 | EU166941 | EU166941 | EU166941 |
| Coraciiformes | Momotidae | *Momotus momota* | Blue-crowned motmot | EU167058 | EU167004 | EU166942 | EU166942 | EU166942 |
| Coraciiformes | Phoeniculidae | *Phoeniculus purpureus* | Green wood hoopoe | EU167067 | EU167012 | EU166943 | EU166943 | EU166943 |
| Coraciiformes | Upupidae | *Upupa epops* | Eurasian hoopoe | EU167086 | EU167030 | EU166944 | EU166944 | EU166944 |
| Cuculiformes | Coccyzidae | *Coccyzus erythropthalmus* | Black-billed cuckoo | AF082048 | AY274015 | AY274062 | AY274062 | AY274062 |
| Cuculiformes | Crotophagidae | *Crotophaga ani* | Smooth-billed ani | AY274035 | AY274016 | AY274063 | AY274063 | AY274063 |
| Cuculiformes | Cuculidae | *Cuculus canorus* | Common cuckoo | AY274034 | AY274013 | AY274060 | AY274060 | AY274060 |
| Cuculiformes | Neomorphidae | *Neomorphus geoffroyi* | Rufous-vented ground cuckoo | AY274036 | AY274017 | AY274064 | AY274064 | AY274064 |
| Falconiformes | Accipitridae | *Buteo jamaicensis* | Red-tailed hawk | AY274044 | U83720 | AY274074 | AY274074 | AY274074 |
| Falconiformes | Cathartidae | *Cathartes aura* | Turkey vulture | EU167038 | EU166984 | EU166946 | EU166946 | EU166946 |
| Falconiformes | Falconidae | *Falco peregrinus* | Peregrine falcon | AF090338 | AF090338 | AF090338 | AF090338 | AF090338 |
| Falconiformes | Pandionidae | *Pandion haliaetus* | Osprey | EU167063 | EU167008 | EU166947 | EU166947 | EU166947 |
| Falconiformes | Sagittaridae | *Sagittarius serpentarius* | Secretary bird | EU167078 | AJ604483 | EU166948 | EU166948 | EU166948 |
| Gaviiformes | Gaviidae | *Gavia adamsi* | Yellow-billed loon | EU167048 | EU166994 | EU166951 | EU166951 | EU166951 |
| Gaviiformes | Gaviidae | *Gavia arctica* | Arctic loon | AY139633 | AY139635 | AY139634 | AY139634 | AY139634 |
| Gaviiformes | Gaviidae | *Gavia immer* | Common loon | EU167047 | EU166993 | EU166950 | EU166950 | EU166950 |
| Gaviiformes | Gaviidae | *Gavia pacifica* | Pacific loon | EU167049 | EU166995 | EU166952 | EU166952 | EU166952 |
| Gaviiformes | Gaviidae | *Gavia stellata* | Red-throated loon | EU167050 | EU166996 | EU166953 | EU166953 | EU166953 |
| Gruiformes | Gruidae | *Grus canadensis* | Sandhill crane | EU167051 | EU166997 | EU166954 | EU166954 | EU166954 |
| Gruiformes | Otididae | *Eupodotis senegalensis* | White-bellied bustard | EU167062 | EU167007 | EU166955 | EU166955 | EU166955 |
| Gruiformes | Rallidae | *Fulica americana* | American coot | EU167074 | EU167019 | EU166956 | EU166956 | EU166956 |
| Musophagiformes | Musophagidae | *Crinifer piscator* | Western grey plantain-eater | AY274040 | AY274021 | AY274068 | AY274068 | AY274068 |
| Musophagiformes | Musophagidae | *Musophaga violacea* | Violet turaco | AY274039 | AY274020 | AY274067 | AY274067 | AY274067 |
| Opisthocomiformes | Opisthocomidae | *Opisthocomus hoazin* | Hoatzin | AY274027 | AY274048 | AF076363 | AF076363 | AF076363 |
| Passeriformes | Acanthisittidae | *Acanthisitta chloris* | Rifleman | AY325307 | AY325307 | AY325307 | AY325307 | AY325307 |
| Passeriformes | Corvidae | *Corvus frugilegus* | Rook | Y18522 | Y18522 | Y18522 | Y18522 | Y18522 |
| Passeriformes | Dendrocolaptidae | *Lepidocolaptes wagleri* | Scaled woodcreeper | EU167041 | EU166987 | EU166957 | EU166957 | EU166957 |
| Passeriformes | Eurylaimidae | *Smithornis sharpei* | Grey-headed broadbill | AF090340 | AF090340 | AF090340 | AF090340 | AF090340 |
| Passeriformes | Formicariidae | *Grallaria squamigera* | Undulated antpitta | AY139636 | AY139638 | AY139637 | AY139637 | AY139637 |
| Passeriformes | Furnariidae | *Cranioleuca baroni* | Baron’s spinetail | EU167045 | EU166991 | EU166958 | EU166958 | EU166958 |
| Passeriformes | Laniidae | *Lanius collurio* | Red-backed shrike | EU167054 | EU167000 | EU166959 | EU166959 | EU166959 |
| Passeriformes | Motacillidae | *Motacilla alba* | White wagtail | EU167059 | EU167005 | EU166960 | EU166960 | EU166960 |
| Passeriformes | Paridae | *Parus major* | Great tit | EU167064 | EU167009 | EU166961 | EU166961 | EU166961 |
| Passeriformes | Picnonotidae | *Hypsipetes amaurotis* | Brown-eared bulbul | EU167068 | EU167013 | EU166962 | EU166962 | EU166962 |
| Passeriformes | Sylviidae | *Phylloscopus occipitalis* | Western crowned-warbler | XXXXXX | XXXXXX | XXXXXX | XXXXXX | XXXXXX |
| Passeriformes | Thraupidae | *Hemispingus frontalis* | Oleaginous hemispingus | AY139639 | XXXXXX | AY139640 | AY139640 | AY139640 |
| Passeriformes | Turdidae | *Catharus guttatus* | Hermit thrush | XXXXXX | XXXXXX | XXXXXX | XXXXXX | XXXXXX |
| Passeriformes | Tyrannidae | *Sayornis phoebe* | Eastern phoebe | AF536744 | AF536741 | AF536747 | AF536747 | AF536747 |
| Passeriformes | Viduidae | *Vidua chalybeata* | Village indigobird | AF090341 | AF090341 | AF090341 | AF090341 | AF090341 |
| Passeriformes | Zosteropidae | *Zosterops japonica* | Japanese white-eye | AY136569 | XXXXXX | XXXXXX | AY136599 | XXXXXX |
| Pelecaniformes | Fregatidae | *Fregata aquila* | Ascension frigatebird | EU167044 | EU166990 | EU166963 | EU166963 | EU166963 |
| Pelecaniformes | Phaethontidae | *Phaethon rubricauda* | Red-tailed tropicbird | EU167065 | EU167010 | EU166964 | EU166964 | EU166964 |
| Pelecaniformes | Phalacrocoracidae | *Phalacrocorax pelagicus* | Pelagic cormorant | EU167066 | EU167011 | EU166965 | EU166965 | EU166965 |
| Pelecaniformes | Sulidae | *Sula dactylatra* | Masked booby | XXXXXX | XXXXXX | XXXXXX | XXXXXX | XXXXXX |
| Phoenicopteriformes | Phoenicopteridae | *Phoenicopterus ruber* | Greater flamingo | AY274045 | U83714 | AY274075 | AY274075 | AY274075 |
| Piciformes | Bucconidae | *Malacoptila semicincta* | Semicollared puffbird | EU167035 | EU166981 | EU166966 | EU166966 | EU166966 |
| Piciformes | Capitonidae | *Trachyphonus usambiro* | Usambiro barbet | EU167036 | EU166982 | EU166967 | EU166967 | EU166967 |
| Piciformes | Galbulidae | *Galbula pastazae* | Coppery-chested jacamar | EU167046 | EU166992 | EU166968 | EU166968 | EU166968 |
| Piciformes | Lybiidae | *Pogoniulus pusilus* | Red-fronted tinkerbird | EU167056 | EU167002 | EU166971 | EU166971 | EU166971 |
| Piciformes | Picidae | *Colaptes auratus* | Northern flicker | XXXXXX | XXXXXX | XXXXXX | XXXXXX | XXXXXX |
| Piciformes | Ramphastidae | *Andigena cuculata* | Hooded mountain-toucan | EU167076 | EU167021 | EU166970 | EU166970 | EU166970 |
| Piciformes | Ramphastidae | *Selenidera reinwardtii* | Golden-collared toucanet | EU167075 | EU167020 | EU166969 | EU166969 | EU166969 |
| Podicipediformes | Podicipedidae | *Podiceps caspicus* | Eared grebe | EU167071 | EU167016 | EU166973 | EU166973 | EU166973 |
| Podicipediformes | Podicipedidae | *Podilymbus podiceps* | Pied-billed grebe | EU167070 | EU167070 | EU166972 | EU166972 | EU166972 |
| Procellariiformes | Diomedeidae | *Diomedea melanophrys* | Black-browed albatross | AY158677 | AY158677 | AY158677 | AY158677 | AY158677 |
| Procellariiformes | Diomedeidae | *Diomedea nigripes* | Black-footed albatross | EU167042 | EU166988 | EU166974 | EU166974 | EU166974 |
| Procellariiformes | Procellariidae | *Calonectris diomedea* | Cory’s shearwater | AY139624 | AY139626 | AY139625 | AY139625 | AY139625 |
| Procellariiformes | Procellariidae | *Pterodroma brevirostris* | Kerguelen petrel | AY158678 | AY158678 | AY158678 | AY158678 | AY158678 |
| Procellariiformes | Procellariidae | *Pterodroma hasitata* | Black-capped petrel | EU167072 | EU167017 | EU166975 | EU166975 | EU166975 |
| Psittaciformes | Psittacidae | *Nandayus nenday* | Nanday parakeet | AY274038 | AY274019 | AY274066 | AY274066 | XXXXXX |
| Psittaciformes | Psittacidae | *Neophema elegans* | Elegant parrot | AY274037 | AY274018 | AY274065 | AY274065 | AY274065 |
| Psittaciformes | Psittacidae | *Strigops habroptilus* | Kakapo | AY309456 | AY309456 | AY309456 | AY309456 | AY309456 |
| Sphenisciformes | Spheniscidae | *Aptenodytes patagonicus* | King penguin | AY139621 | AY139623 | AY139622 | AY139622 | AY139622 |
| Sphenisciformes | Spheniscidae | *Eudyptes.chrysocome* | Rockhopper penguin | AY139630 | AY139632 | AY139631 | AY139631 | AY139631 |
| Sphenisciformes | Spheniscidae | *Eudyptula minor* | Little penguin | AF362763 | AF362763 | AF362763 | AF362763 | AF362763 |
| Strigiformes | Strigidae | *Asio otus* | Long-eared owl | AY274022 | AF08206 | AY274069 | AY274069 | AY274069 |
| Strigiformes | Strigidae | *Ninox novaeseelandiae* | Morepork | AY309457 | AY309457 | AY309457 | AY309457 | AY309457 |
| Strigiformes | Tytonidae | *Tyto alba* | Barn owl | EU167085 | EU167029 | EU166976 | EU166976 | EU166976 |
| Trogoniformes | Trogonidae | *Harpactes ardens* | Philippine trogon | U94810 | U94796 | XXXXXX | XXXXXX | XXXXXX |
| Trogoniformes | Trogonidae | *Trogon curucui* | Blue-crowned trogon | AY274031 | AY274009 | AY274056 | AY274056 | XXXXXX |
